# Tolerant Molecular Engineering for High‐Rate and Ultra‐Long Cycle Life Zinc Anode

**DOI:** 10.1002/advs.202508628

**Published:** 2025-07-02

**Authors:** Chenyang Zhao, Zeping Liu, Yu Zhang, Pengyu Wang, Man Qi, Zhikun Guo, Zeen Wu, Xin Zhang, Xingyuan Lu, Jiayin Yuan, Naiqing Zhang

**Affiliations:** ^1^ The State Key Laboratory of Urban‐rural Water Resources and Environment School of Chemistry and Chemical Engineering Harbin Institute of Technology Harbin 150001 China; ^2^ Department of Chemistry Stockholm University Stockholm 10691 Sweden

**Keywords:** stability, zinc anode, zinc ion batteries

## Abstract

Aqueous zinc ion batteries have emerged as a promising technology for grid‐scale energy storage due to their low cost and high safety. However, dendrites and side reactions at the zinc anode severely deteriorate their cycle stability. Herein, to mitigate these issues, a molecular tolerance mechanism, inspired by cell membranes in extreme plants, is employed to engineer the zinc anode surface. The molecular layer enhances both the desolvation process and transport rate of zinc ions, thereby suppressing the dendrite formation. The interfacial side reactions caused by excessive water molecules in solvated ions are practically minimized as well, thus the zinc anode turns more durable and robust under high‐rate conditions. Specifically, at a moderate current density of 1 mA cm^−2^@1 mAh cm^−2^, the cycle stability of the zinc anode is improved to 8800 h, and up to 1100 h at a high current density of 10 mA cm^−2^@5 mAh cm^−2^. This work offers valuable insights into interfacial design for developing zinc ion batteries with long cycle life and fast‐charging capabilities.

## Graphical abstract

A tolerant molecular engineering is proposed to greatly improve the stability of the zinc anode. This molecule enhances the conductivity of the electrolyte by reconstructing the hydrogen bonding network and accelerates ion transfer at the interface, resulting in uniform zinc stripping/plating. It also self‐assembles into a tolerant molecular layer on the zinc anode, efficiently maintaining the interfacial stability by suppressing corrosion and side reactions.

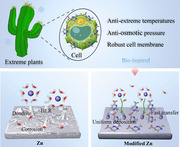

## 1 Introduction

Aqueous zinc ion batteries (AZIBs) have been rapidly developed in the past decade and are attracting great interest in the large‐scale energy storage market, due to the abundant resource and low cost of zinc, high theoretical energy density, and simple assembly under an atmospheric environment.^[^ ^1^, ^2^, ^3^ ^]^ Besides, in terms of flammability, AZIBs use aqueous electrolytes of higher safety than organic counterparts used in most rechargeable batteries, such as lithium or sodium ion batteries. Using metal zinc anodes in AZIBs is of great importance in achieving high energy density because of its high capacity of 820 mAh g^−1^ and lower redox potential of −0.76 V versus the standard hydrogen electrode.^[^ ^4^, ^5^, ^6^ ^]^ However, the cycle life of zinc anode, especially under high current density and large capacity, faces challenges because of the destructive dendrite growth of zinc upon discharge/charge cycles, and the side reactions at the zinc surface that reduce zinc utilization efficiency and increase resistance.^[^ ^7^, ^8^, ^9^ ^]^ Hence, mitigation of dendrite and side reaction issues in zinc anode under various conditions is a crucial step toward the practical application of AZIBs.^[^ ^10^, ^11^ ^]^

So far, active strategies have been proposed to overcome the aforementioned limitations for better stabilizing zinc anodes, such as constructing artificial interface layers,^[^ ^4^, ^12^ ^]^ designing separators,^[^ ^13^, ^14^ ^]^ and introducing electrolyte additives.^[^ ^15^, ^16^, ^17^ ^]^ Among them, electrolyte additives hold promise in operational simplicity, high efficiency, and ease of scalability for the practical production of AZIBs. Although progress has been made in the search for electrolyte additives, most molecules interact weakly with the zinc anode.^[^ ^18^ ^]^ They typically form adsorption molecular layers through van der Waals forces, which results in poor interfacial stability at the electrolyte/anode interface, not to mention the decreased ion conductivity in the electrolyte, to hinder the efficient migration of Zn^2+^. Especially under high‐rate conditions, the interface worsens as a large amount of solvated water molecules disrupts the molecular layer, raising the risk of interfacial failure. Therefore, it is urgent to explore new additive molecules capable of forming robust and stable interfacial layers, without sacrificing ion transport rate.

In nature, extreme plants use their cell membranes to withstand a salty environment, strong solar light, and high or low‐temperature conditions, thus maintaining cellular activity. Among them, the Pyrostatin B molecule (2‐methyl‐1,4,5,6‐tetrahydropyrimidin‐3‐ium‐6‐carboxylate, termed “PyrB”), with its polarized electrostatic potential, can protect the interface between phospholipids and proteins, stabilize membrane structure under extreme conditions, and accommodate high osmotic pressure on cell membranes.^[^ ^19^ ^]^

**Scheme 1:**
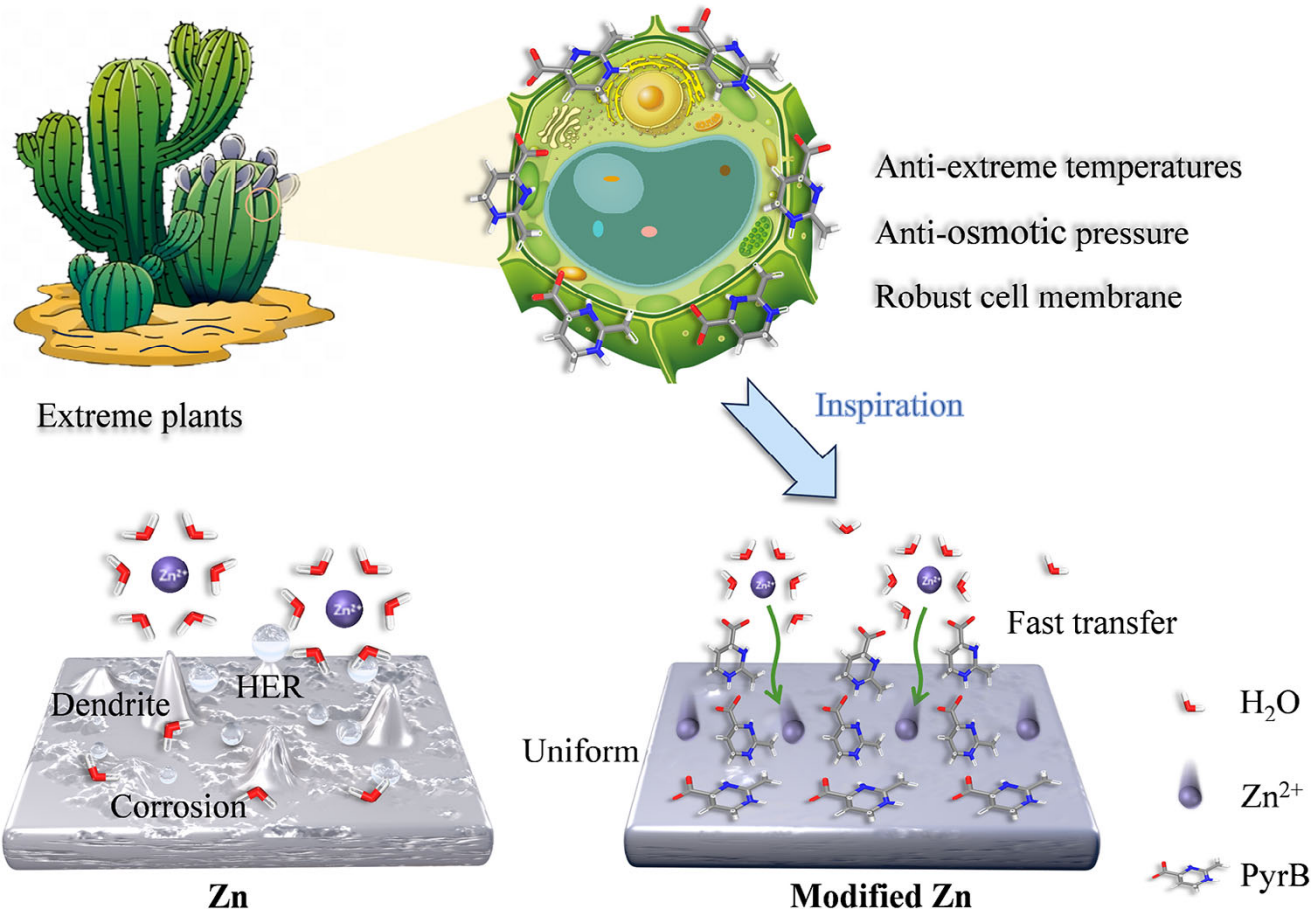
Illustration of the construction of the tolerant molecular layer on the zinc anode, inspired by the stable cell membrane in extreme plants.

Inspired by the tolerant mechanism of the cell membrane under extreme conditions (**Scheme** **1**), we shaped a similar interfacial environment on the zinc anode by adding such a molecule into the aqueous electrolyte. This additive not only self‐assembles into a tolerant molecular layer on the surface of the zinc anode through strong electrostatic interactions, but also boosts ion conduction of the electrolyte by regulating the hydrogen bonding network. Additionally, the modified interface protects the zinc anode from erosion caused by a large number of active water molecules from solvation ions under high‐rate conditions. Consequently, the symmetric zinc cells using this tolerant molecule as an additive can operate steadily for 8800 h at 1 mA cm^−2^ @1 mAh cm^−2^. Even at a high current density of 10 mA cm^−2^@5 mAh cm^−2^, it delivers a cycle life of 1100 h, fully verifying the efficiency of PyrB in improving the lifetime of zinc anodes under high‐rate conditions. PyrB works equally efficiently in both Zn||MnO_2_ and Zn||vanadium oxide full cells.

## 2 Results and Discussion

**Figure 1:**
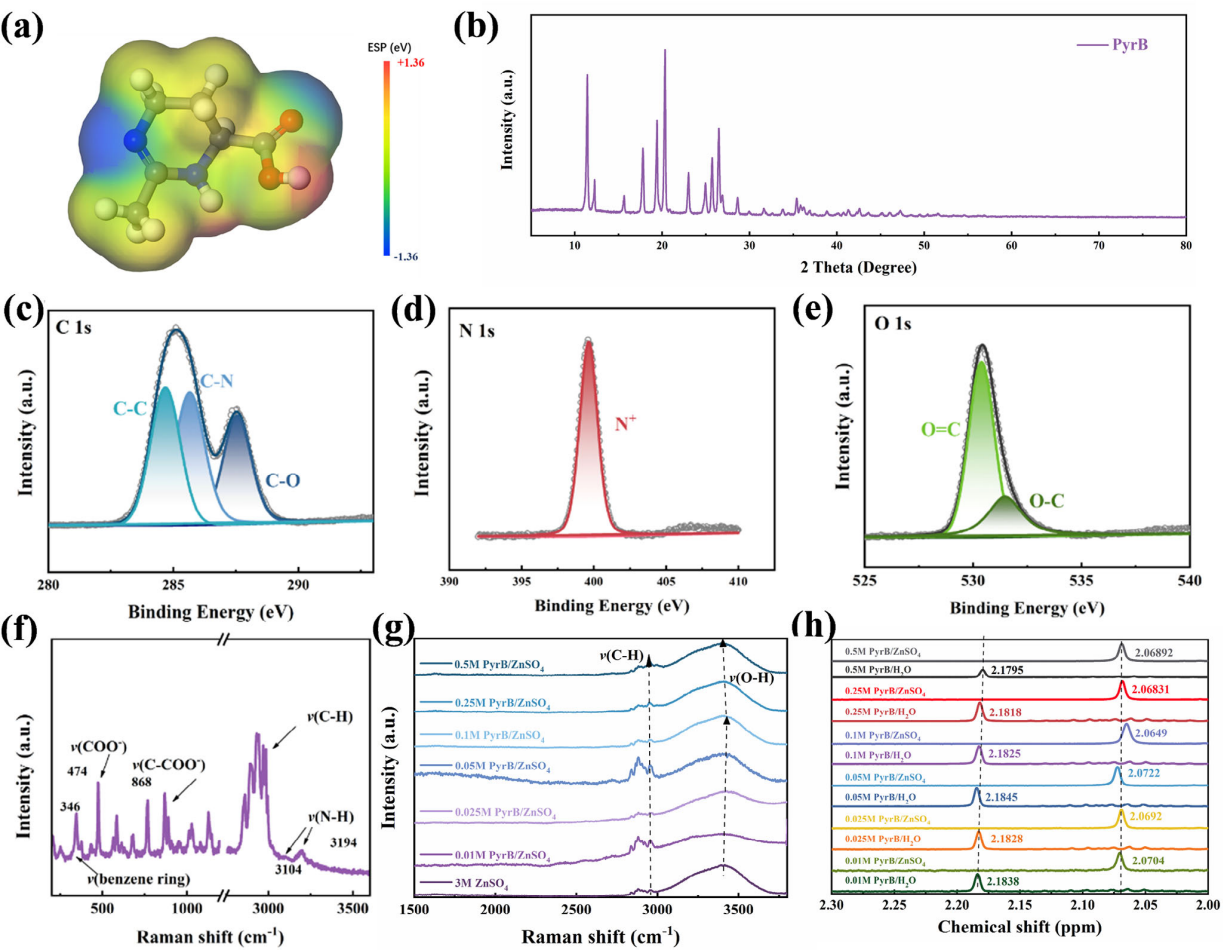
a) Image of the electrostatic potential distribution in PyrB. b) XRD pattern of PyrB. c–e) XPS spectra of C 1s, N 1s, and O 1s for PyrB, respectively. f) Raman spectrum of PyrB. g) Raman spectra of ZnSO_4_ electrolytes at different PyrB concentrations. h) ^1^H NMR spectra of ZnSO_4_ electrolytes at different PyrB concentrations.

PyrB exists in the extreme plant cell membranes. It maintains the cell's structural integrity and activity by mitigating the damage caused by extreme conditions. The strong affinity of PyrB to water molecules, in particular, can minimize the impact of high osmotic pressure on membrane structure.^[^ ^20^ ^]^ Analogously, this character can help build up a “protective cover” on the zinc anode for suppressing the activity of solvation water and maintaining the electrode/electrolyte interface (Scheme 1). Specifically, PyrB molecules can retain water well (Figure S1, Supporting Information) and strongly bind H_2_O to suppress the aquatic corrosion to the zinc anode. The presence of carboxyl groups in PyrB provides migration channels for Zn^2+^, enabling higher rate performance. The electrostatic potential diagram of the PyrB molecule is calculated, as shown in **Figure** **1**a. Compared with O on C═O of the carboxyl group, the N species catches a higher electrostatic potential, so that it becomes a reasonable site in PyrB to adsorb the surface of the zinc anode. The calculated adsorption energy of PyrB on zinc further supports a strong interaction compared with other organic molecules (Figure S2, Supporting Information).

Depending on the external environment and crystallization state, PyrB adopts either a dihydrate or anhydrate form, by varying the arrangement of its carboxyl group and structural stability. Previous studies clarified that the dihydrate form is metastable and readily converted to the thermodynamically stable anhydrate form. The crystalline structure of PyrB was analyzed by X‐ray diffraction (XRD) tests in Figure 1b. The diffraction peaks match the PyrB anhydrate form,^[^ ^19^ ^]^ indicating no H_2_O molecules in PyrB before its addition into the electrolyte. Next, X‐ray photoelectron spectroscopy (XPS) was used to analyze its atoms’ chemical environment. As shown in Figure 1c,e, the elements of C, N, and O in PyrB are detected. The bonding types of C, N, and O are C─C, C─N, C═O, and C─O in PyrB. Among them, three peaks of C 1s appear at 284.6, 285.7, and 287.5 eV, corresponding to C─C, C─N, and C─O bonds, respectively. The N^+^─C peak at 399.6 eV in the N 1s spectrum indicates the presence of the C─N bond in PyrB. Two peaks of O 1s at 530.4 and 531.4 eV, correspond to the C═O and C─O bonds of the carboxylic acid group of PyrB.^[^ ^21^ ^]^ Besides, the bands of *v*(COO─), *v*(C‐COO─), *v*(C─H) and *v*(N─H) located at 474, 868, 2800—3000, and 3194 cm^−1^ respectively, in PyrB's Raman spectrum (Figure 1f) confirm the presence of amino and carboxyl groups in PyrB.

The interaction between PyrB and the aqueous ZnSO_4_ electrolyte was first discussed through Raman spectroscopy. As shown in Figure 1g, the O─H stretching vibration of H_2_O occurs at 3100–3700 cm^−1^. As the content of PyrB increases from 0.01 to 0.1 m, the O─H stretching vibration band at 3418 cm^−1^ shifts to a higher wavenumber, meaning the rising vibrational energy required for the transition. This can be explained by the disruption of the hydrogen bond network in the original aqueous electrolyte when PyrB is added.^[^ ^22^ ^]^ However, at a PyrB concentration (*C_PyrB_*) above 0.1 M, the O‐H stretching vibration band starts shifting to a lower wavenumber, meaning a facilitated vibration transition. It can be attributed to the reconstruction of the hydrogen bond network between electrolyte and PyrB molecules due to the agglomeration of PyrB at high concentrations. Hence, 0.1 m was chosen as an appropriate concentration of electrolyte to reach an optimal ion transfer in the disrupted hydrogen bond network. The effect of PyrB in the electrolyte was further investigated by the ^1^H nuclear magnetic resonance (^1^H NMR) spectroscopy. In comparison with the pure PyrB aqueous solution (Figure 1h), the addition of PyrB to the ZnSO_4_ electrolyte will up‐shift the proton signal (at ≈2.18 ppm) of the PyrB molecule (H atom at methylene adjacent to carboxyl). This shift is a sign of disruption of hydrogen bond networks in a mixture of PyrB, ZnSO_4_, and H_2_O, which suppressed the electron density of PyrB and weakened its shielding effect to this proton. The solution at 0.1 m PyrB and 3 m ZnSO_4_ shows the largest up‐shift, representing the most severe hydrogen bond disruption under this condition. This phenomenon is consistent with results observed from the Raman spectra. It can be inferred that PyrB can seize H_2_O molecules via forming hydrogen bonds and disturb the continuous hydrogen bond network among H_2_O molecules, inhibiting the dynamics of water molecules. Different from the state in which Zn^2+^ is encapsulated by six H_2_O ligands in the pure ZnSO_4_ electrolyte (Figure S3a, Supporting Information), the attraction of PyrB molecules to Zn^2+^‐coordinated H_2_O can break the balance between H_2_O molecules and Zn^2+^ (Figure S3b, Supporting Information). Ionic conductivity test results in Figure S4 (Supporting Information) indicate the optimal amount of PyrB in electrolyte. With continuous addition of PyrB, the ionic conductivity gradually rises and peaks at *C_PyrB_* = 0.1 m. The ionic conductivity of 0.1 m PyrB in 3.0 m ZnSO_4_ (measured as 0.178 S cm^−1^) is even higher than the original 3.0 m ZnSO_4_ electrolyte (0.144 S cm^−1^). Clearly, electrolytes containing PyrB accelerate the transfer of zinc ions.

**Figure 2:**
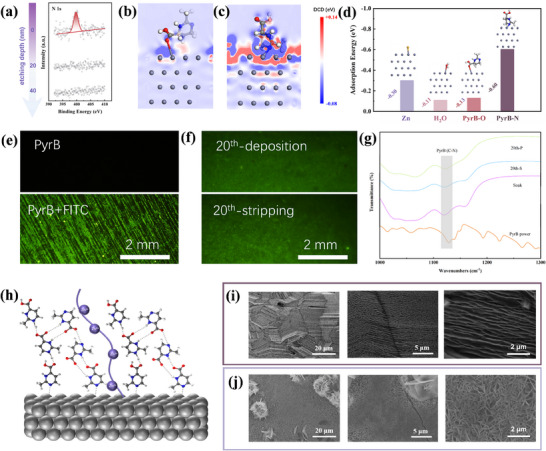
a) XPS spectra of N 1s of PyrB@Zn at different depths of etching. The sliced 2D contour map of different charge densities of b) PyrB‐O and c) PyrB‐N with Zn(002) at the interface. d) Adsorption energies of Zn^2+^, H_2_O, PyrB‐O, and PyrB‐N on the surface of Zn (002). e) Fluorescence labeling experiment of PyrB@Zn before cycle tests. f) Fluorescence photo of PyrB@Zn after 20 cycles. g) Infrared spectroscopy testing of electrodes after soaking and cycling. h) Diagram of PyrB adsorption on Zn metal surface and Zn^2+^ transport pathway. i,j) SEM images of zinc deposition morphology at 1.0 mA cm^−2^ and 5.0 mAh cm^−2^ on (i) PyrB@Zn, (j) bare Zn anode.

The molecular layer formed by adsorptive assembly of PyrB on zinc anode (termed PyrB@Zn) is verified by ex situ XPS measurement via stepwise surface etching, as shown in **Figure** **2**a. The high resolution of N 1s spectrum of PyrB@Zn before etching is consistent with that of the pristine PyrB molecule, confirming absorption of PyrB on. Upon the etching, the N 1s peak disappears after 20 min. Given the etching rate of 1 nm min^−1^, the thickness of PyrB@Zn is expected up to 20 nm. The ion migration number ($t_{Zn^{2+}}$) is calculated based on the results from chronoamperometry (CA) and electrochemical impedance spectroscopy (EIS) tests^[^ ^23^, ^24^, ^25^ ^]^ (Figure S5, Supporting Information). The $t_{Zn^{2+}}$ of PyrB@Zn is 0.83, much higher than 0.46 of the bare zinc anode, due to the effective transport of Zn^2+^ by carboxyl groups. The improved $t_{Zn^{2+}}$ will contribute to the uniform zinc deposition/stripping during the charge/discharge processes, maintaining the stability of the zinc anode. Figure S6 (Supporting Information) displays the initial CA curves under −150 mV to investigate the ion transfer difference. PyrB@Zn enters a stable three‐dimensional diffusion process after only 44 s of planar diffusion. In contrast, the current density of the bare zinc anode continues to fluctuate after 50 s, and 2D diffusion mass transfer is still present on the bare zinc anode.^[^ ^26^ ^]^

The adsorption mechanism of PyrB on different crystal planes of the Zn anode is clarified through a theoretical study. Figure 2b,c and Figure S7 (Supporting Information) reveal the sliced 2D contour map at different charge densities of various sites of PyrB@Zn. Compared with the oxygen adsorption site (PyrB‐O, Figure 2b), the nitrogen one (PyrB‐N, Figure 2c) shows strong interaction with Zn and the corresponding charge transfer at the interface, suggesting that PyrB molecules prefer adsorption on the zinc anode through the nitrogen site. The difference in binding energy between different molecules and Zn are shown in Figure 2d. The binding energies of H_2_O molecules and Zn^2+^ on the Zn (002) surface are −0.11 and −0.30 eV, respectively. PyrB‐O and PyrB‐N adsorption sites provide binding energies of −0.13 and −0.60, respectively. The strongest binding energy of −0.60 eV prioritizes PyrB‐N sites toward Zn. Thus, PyrB first adsorbs to the zinc surface through the nitrogen sites prior to H_2_O and Zn^2+^, preventing the zinc anode from corrosion by the latter two. Further fluorescence labeling experiments intuitively demonstrate that PyrB has a strong adsorption ability on zinc surfaces (Figure 2e). The adsorption interaction between PyrB and fluorescein isothiocyanate enables the green fluorescence labeling of PyrB on the zinc anode. After 20 cycles, the electrode still exhibits strong fluorescence signals (Figure 2f), indicating the long‐term effectiveness of the adsorption molecular layer. The infrared spectroscopy test (Figure 2g) verifies stable adsorption of PyrB molecules on the zinc anode after 20th stripping (20th‐S) and plating(20th‐S). Figure 2h illustrates the adsorption layer structure of PyrB on zinc surface. The imine group at the end of PyrB is adsorbed on the zinc surface, preventing H_2_O molecules in the solvated Zn^2+^ ions from contacting the Zn anode. The carboxyl group at the other end of PyrB can attract Zn^2+^ to facilitate ion transfer.^[^ ^27^ ^]^ The interface stability of the adsorption layer can be further supported by the molecular orbital theory.^[^ ^28^ ^]^ As shown in Figure S8 (Supporting Information), PyrB has a higher occupied molecular orbital (−5.42 eV) than H_2_O (−7.16 eV), implying that PyrB rather than H_2_O is more likely to lose electrons when adsorbed on the surface of Zn. During the adsorption process, electrons tend to migrate from PyrB to Zn to build up strong interactions to ensure a robust adsorption layer. The effect of the PyrB adsorbed layer on the zinc deposition behavior was first investigated by scanning electron microscope (SEM). As shown in Figure 2i,j, after deposition at a current density of 1.0 mA cm^−2^ within an areal capacity of 5.0 mAh cm^−2^, the surface of the bare zinc anode becomes rough. In contrast, the deposited zinc on PyrB@Zn is relatively flat and dense without the formation of dendrite. XRD analysis was performed on the pure Zn foil and the PyrB@Zn electrode after zinc deposition (Figure S9, Supporting Information). No obvious difference in the crystal orientation was found between both, indicating an undisturbed deposition process of the zinc anode through the tight packing. The separators after the test are checked in Figure S10 (Supporting Information). There is obviously deposited zinc in the separator of the symmetric Zn cell without PyrB, while no deposition of zinc when PyrB is added to the electrolyte. After a long period of cycling, the surface of the zinc electrode becomes rough and dendrites further accumulate (Figure S11, Supporting Information). However, for PyrB@Zn, due to effective molecular protection, it maintains its stable state even after 50 cycles.

**Figure 3:**
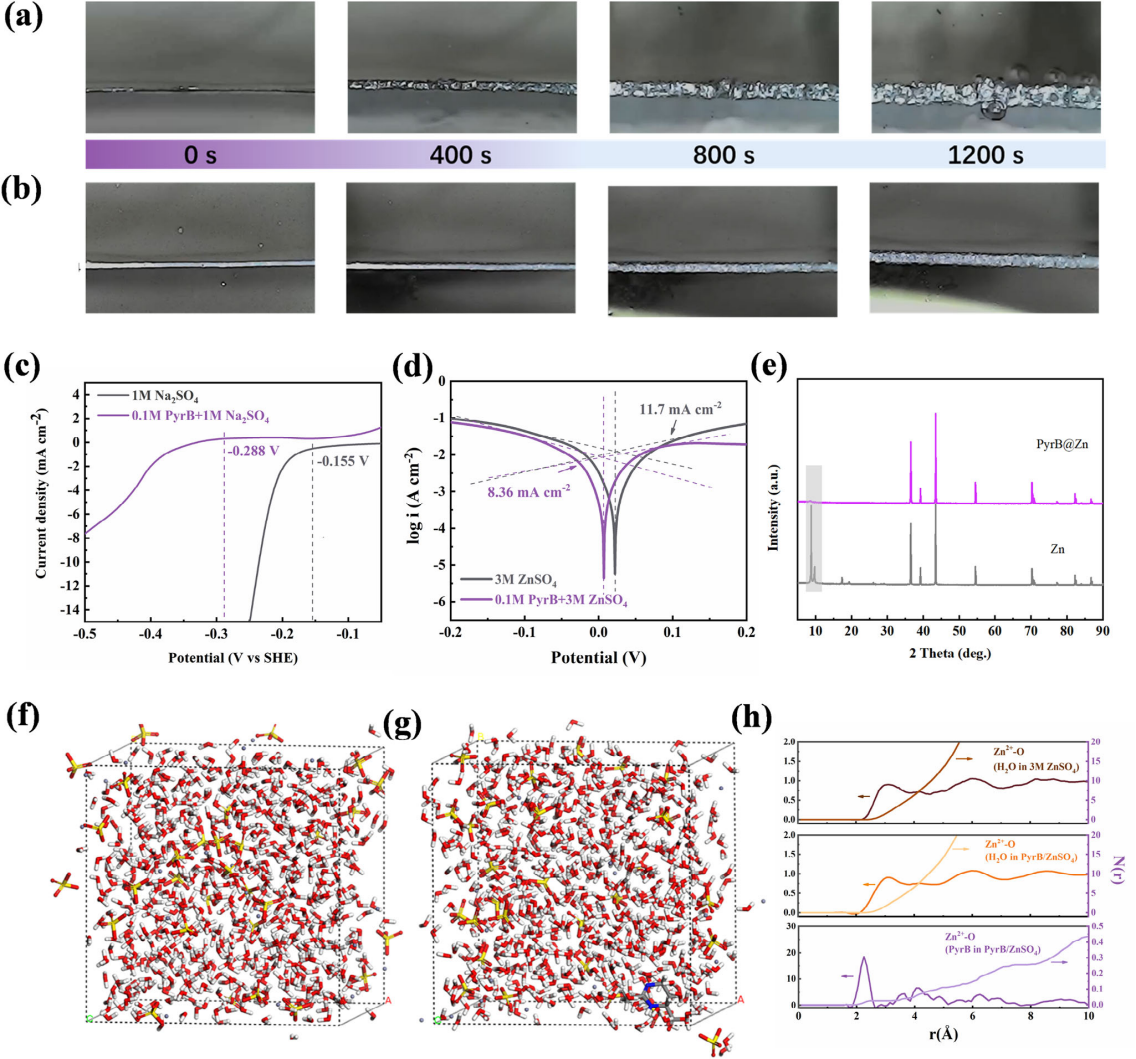
The optical photographs of in situ monitoring of zinc deposition morphology a) bare Zn; b) PyrB@Zn. c) Linear sweep voltammetry curves of Zn in the 1 m Na_2_SO_4_ and 0.1 m PyrB/1 m Na_2_SO_4_ aqueous electrolyte; d) Tafel curves of Zn anode with or without PyrB layer; e) XRD results of zinc foil soaked in different electrolytes at 60 °C for two days, The MD results of f) 3 m ZnSO_4_ and g) 0.1 m PyrB/3 m ZnSO_4_; h) The radial distribution functions and coordination number between Zn^2+^ and O of solvents.

To directly observe the difference of zinc deposition with the PyrB molecular layer on zinc, in situ optical microscopy was used for the real‐time tracking of the morphological evolution during the zinc deposition process.^[^ ^29^ ^]^ As shown in **Figure** **3**a, at a current density of 40.0 mA cm^−2^, sharp protrusions and H_2_ bubbles rapidly appear on the surface of the bare Zn anode. When the plating reaches 800 s, the surface is covered by a large number of zinc dendrites and bubbles. In contrast, the PyrB@Zn maintains a relatively flat deposition morphology throughout the entire process without the observation of H_2_ bubbles or zinc dendrites. The PyrB molecular layer enables uniform zinc deposition even after an extended deposition time of 1200 s, with no sharp protrusions or hydrogen bubbles observed on the surface (Figure 3b). After confirming the inhibitory effect of the PyrB molecular layer on the growth of dendrites, the effect on hydrogen evolution and related side reactions was investigated. As shown in Figure 3c, the hydrogen evolution overpotential of the PyrB@Zn is higher than that of the bare zinc anode at the same current density. The inflection point potential of the PyrB@Zn at the beginning of hydrogen evolution is −0.288 V, while that of the bare zinc anode is ‐0.155 V, thus, PyrB can inhibit the hydrogen evolution. In addition, the bulging degree of the symmetric Zn cell after 50 cycles (at a current density of 10.0 mA cm^−2^ and areal capacity of 5.0 mAh cm^−2^) is displayed in Figure S12 (Supporting Information). Bare zinc cell shows significant bulging due to a severe hydrogen evolution reaction during the cycles. In comparison, the PyrB@Zn cell remains intact after cycles, which also suggests that the hydrogen evolution reaction is significantly suppressed. Figure 3d compares the corrosion curves of different zinc anodes. The corrosion current of the PyrB@Zn (2.27 mA cm^−2^) is lower than the bare zinc anode (3.18 mA cm^−2^), which is consistent with the protection effect from PyrB molecular layers.^[^ ^30^ ^]^ The soaking experiment verifies that PyrB molecules effectively inhibit the occurrence of side reactions, particularly for the strong byproduct peak exhibited by bare zinc (Figure 3e).

In the radial distribution function calculated by molecular dynamics (MD), the main solvation structure of Zn^2+^ in aqueous solution is $\mathrm{Zn}{\left(H_{2}O\right)}_{6}^{2+}$ with an average coordination distance of 2.95 Å and a coordination number of 5.83 in 3 m ZnSO_4_ electrolyte (Figure 3f). The addition of PyrB weakens the Zn─O (H_2_O) coordination number to 5.16 (Figure 3g). The Zn‐O (PyrB) coordination structure appears at a coordination distance of 2.25 Å in the radial distribution function. Since the concentration of PyrB is 0.1 m, only 1/30 of ZnSO_4_, thus the calculated coordination number is only 0.1 (Figure 3h). These MD simulations reveal that PyrB molecules preferentially enter the solvation shell of Zn^2+^, forming a Zn^2+^‐PyrB complex. This interaction disrupts the solvation structure of Zn^2+^, interferes with the hydrogen bond network among H_2_O molecules, and alters the solvation equilibrium of Zn^2+^. The solvated complex of Zn^2+^ in the presence of PyrB is expected to reduce the interference from active H_2_O molecules, suppressing the hydrogen evolution reaction.^[^ ^31^ ^]^

**Figure 4:**
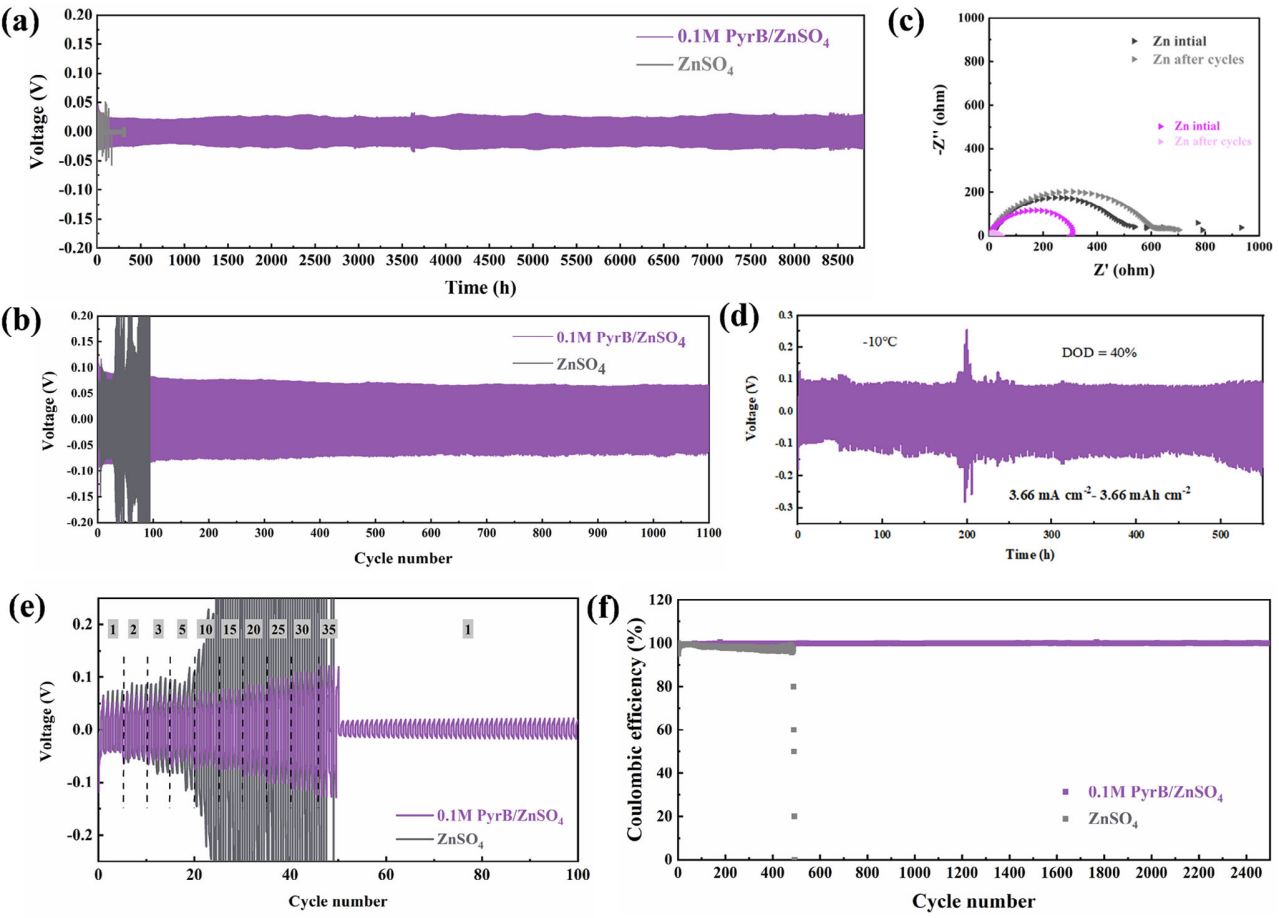
a) Voltage profiles of symmetric cells based on bare Zn foil and PyrB@Zn anodes at 1.0 mA cm^−2^ with a capacity of 1.0 mAh cm^−2^; b) Symmetric cell cycle curve at 10 mA cm^−2^ for 5 mAh cm^−2^; c) EIS plots of Zn||Zn cells of initial state and after cycles; d) Low‐temperature cycling performance of symmetrical cells; e) Rate performance of zinc symmetrical cells; f) CE performance of Zn||Cu cells at current density of 1.0 mA cm^−2^ and areal capacity of 1.0 mAh cm^−2^.

The zinc deposition/stripping cycle was investigated by galvanostatic charge–discharge test in symmetric zinc cells. As shown in **Figures** **4**a and S13 (Supporting Information), at a current density of 1 mA cm^−2^ and an areal capacity of 1 mAh cm^−2^, the bare zinc anode delivers a short circuit at ≈100 h. In contrast, the PyrB@Zn can cycle above 8800 h with a stable hysteresis voltage. The slight fluctuation of voltage at ≈3600 h is due to the disturbances of ambient temperature and the voltage remains stable in the subsequent deposition/stripping process, confirming the effectiveness of PyrB to stabilize the zinc anode. Figure S14 (Supporting Information) compares the cycle stability of symmetric zinc cells at a high rate of 10 mA cm^−2^ at a capacity of 1 mAh cm^−2^. Bare zinc can merely work for 700 cycles, while the PyrB@Zn proceeds constantly for 2500 cycles. When the areal capacity increases to 5 mAh cm^−2^ (Figure 4b), the bare zinc starts to fail after 100 cycles, while the PyrB@Zn retains 1100 stable cycles, 11 times that of the bare zinc. When the rate rises further to 20 mA cm^−2^ (Figure S15, Supporting Information), the bare zinc shows large voltage fluctuation after 47 cycles due to the formation of a large amount of by‐products and dead zinc accumulation under high‐rate.^[^ ^32^ ^]^ In the case of PyrB@Zn, it operates smoothly for 450 cycles without polarization fluctuations, suggesting that the PyrB adsorbed layer also functions well under high‐rate conditions. Under even higher zinc utilization conditions (60%), the PyrB@Zn maintains good cycle performance (Figure S16, Supporting Information), verifying the effectiveness of the tolerant adsorption layer under harsh conditions. EIS data before and after the plating/stripping process of the zinc anode are shown in Figure 4c to investigate the interface behavior.^[^ ^33^, ^34^ ^]^ After cycling for 100 h, bare zinc raises its interfacial charge transfer resistance (R_ct_) to 605 Ω due to the continuous side reactions and generated irregular Zn dendrites during repeated discharge/charge cycles. As for PyrB@Zn, the R_ct_ decreases dramatically to 5 Ω after cycles, indicating the stable molecular layer on the surface of Zn anode and its suppression on the side reactions. Besides, the PyrB@Zn works well at low temperatures (Figure 4d), with a cycling life of over 550 h at −10 °C and a zinc utilization degree of 40%. However, the zinc sulfate electrolyte has solidified and cannot function properly (Figure S17, Supporting Information). Figure 4e displays the rate performance of symmetric Zn cells from 1  to 35 mA cm^−2^. The voltage polarization of the bare zinc increases sharply when reaching the current density of 10.0 mA cm^−2^, resulting in the failure of the cell. In contrast, the symmetric PyrB@Zn cell shows stable hysteresis voltage even at a current density of 35 mA cm^−2^. The hysteresis voltage remains constant when the current density returns to 1 mA cm^−2^. The activation energy fitting was performed through variable temperature impedance (Figure S18, Supporting Information), and the results show that PyrB@Zn delivers a decreased value of 17.25 KJ mol^−1^ compared to bare Zn of 43.23 KJ mol^−1^.^[^ ^35^ ^]^

Next, Zn||Cu half cells were assembled for testing Coulombic efficiency (CE) to evaluate the reversibility of Zn plating/stripping reactions. As shown in Figure 4f, the Coulombic efficiency of the bare Zn||Cu cell decreases significantly after 400 cycles at 1 mA cm^−2^, indicating the failure of Zn||Cu cells. In contrast, the Zn||Cu with PyrB delivers stable CE of ≈99.9% over 2400 cycles. When the current density was increased to 5 mA cm^−2^ (Figure S19, Supporting Information), the PyrB@Zn kept a long cycle stability of 1200 cycles compared with bare Zn of 92 cycles. The cyclic voltammetry (CV) curves of Zn||Cu half cells in Figure S20 (Supporting Information) verify the enhanced Zn plating/stripping reactions from the PyrB molecular layer. Both CV curves present typical cathode loops and anode peaks for the zinc plating and stripping reaction, respectively. For Zn||Cu cell with PyrB molecular layer, the overpotential of Zn^2+^ deposition decreases by 16 mV compared to bare Zn deposition. The lower deposition overpotential indicates the rapid transfer of Zn^2+^ and promotes the uniform deposition of zinc. Moreover, compared to the previous studies (Table S1, Supporting Information),^[^ ^34^, ^36^, ^37^, ^38^, ^39^, ^40^ ^]^ symmetric Zn cells with PyrB are advantageous in cycle stability and rate performance.

**Figure 5:**
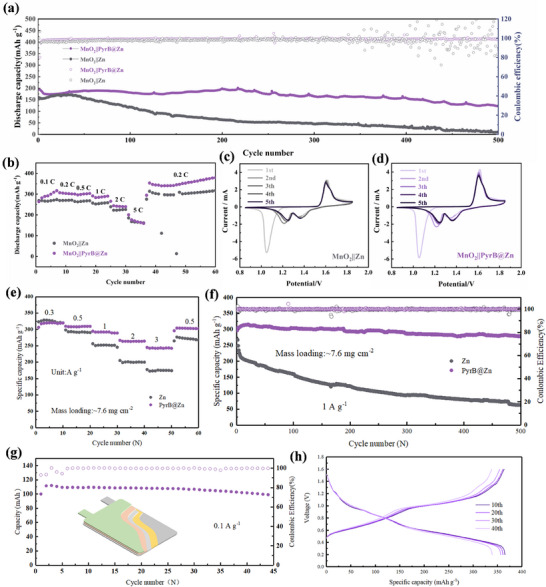
a) Cycle performance of Zn||MnO_2_ and PyrB@Zn||MnO_2_ full cells. b) Rate performance of Zn||MnO_2_ and PyrB@Zn||MnO_2_. Galvanostatic charge/discharge curves of c) Zn||MnO_2_ d) PyrB@Zn||MnO_2_ cells (1C = 308 mA g^−1^, based on MnO_2_). e) Rate performance of Zn||vanadium oxide full cells. f) Long cycle performance of Zn||vanadium oxide full cells under condition 1 A g^−1^. g) Cycling performance of pouch cell with vanadium oxide cathode and corresponding h) voltage capacity curve.

The full cells are next assembled and electrochemical performance tests are carried out. It was first shown by morphology and mapping that manganese oxide and vanadium oxide cathode materials were successfully prepared (Figure S21, Supporting Information). As shown in **Figure** **5**a with the MnO_2_ cathode, the modified zinc cell preserves a discharge capacity of 123.1 mAh g^−1^ after 500 cycles at a current density of 1 A g^−1^. In comparison, the capacity of a bare zinc full cell fades quickly to almost zero. In addition to improving the zinc anode's performance, PyrB also adsorbs onto the surface of the cathode (Figure S22, Supporting Information) and alleviates problems associated with dissolution and distortion of electrode material, thereby cycling better. Under different current densities, PyrB@Zn full cell has a higher discharge capacity than the full cell without PyrB, suggesting the improved rate performance in PyrB@Zn full cells (Figure 5b). The CV curves in Figure 5c,d present two pairs of redox peaks of H^+^/Zn^2+^ embedded and exfoliated from MnO_2_.^[^ ^41^ ^]^ It is noteworthy that the redox peak potential difference of the PyrB@Zn full cell is reduced, indicating a reduction of electrode polarization with better reversibility. In addition, the higher redox peaks in the CV curves of the PyrB@Zn full cell also suggest improved ion storage. Later, a vanadium oxide cathode is evaluated, and PyrB@Zn also exhibits good rate performance (Figure 5e). At the current density of 1 A g^−1^, after 500 cycles, the battery bearing PyrB@Zn still maintains a capacity of 278.6 mAh g^−1^ with a retention rate of 91.5%. In contrast, bare zinc full cells exhibit rapid degradation due to severe dendrite and side reaction issues, with a capacity retention rate of only 21.1% (Figure 5f). Even at −10 °C, PyrB@Zn full cell demonstrates good cycling stability (Figure S23, Supporting Information), fully exhibiting its excellent low‐temperature resistance and rapid kinetic characteristics. Finally, to verify the practical potential of PyrB, a soft pack battery is evaluated (Figure 5g,h), which releases over 100 mAh of capacity and maintains a capacity retention rate of ≈100% after cycles, demonstrating excellent cycling stability.

## 3 Conclusion

In summary, a bio‐inspired tolerant molecular layer was built up from PyrB on the surface of the zinc anode to boost the stability of the zinc anode in rechargeable AZIBs. This layer ensures the interfacial stability by replacing the weak van der Waals forces between traditional organic molecules with strong electrostatic interactions. The experimental data prove that the adsorbed PyrB molecules can accelerate the transfer rate of Zn^2+^ by optimizing the interface transport channel, thereby enabling cycling stability under high‐rate charge/discharge conditions. More importantly, the tolerant molecular layer minimizes the erosion of zinc anode by water molecules. Symmetric cell based on PyrB@Zn grants ultralong cycle life of 8000 h under 1 mA cm^−2^@1 mAh cm^−2^. When the rate increases to 10 mA cm^−2^ and the capacity to 5 mA cm^−2^, the symmetric cell is cycled steadily over 1100 h. The Zn||MnO_2_ full cell under the PyrB protection reserves a specific capacity of 123.1 mAh g^−1^ after 500 cycles at 1 A g^−1^. The Zn||vanadium oxide full cell with PyrB delivers a reversible capacity of 278.6 mAh g^−1^ after 500 cycles at a current density of 1 A g^−1^ at the cathode loading of 7.6 mg cm^−2^. This study offers a facile strategy to establish a protective molecular layer on the anode for developing high‐performance rechargeable metal batteries.

## Conflict of Interest

The authors declare no conflict of interest.

## Supporting information



Supporting Information

## Data Availability

The data that support the findings of this study are available from the corresponding author upon reasonable request.
