# Supplementary material for: Tolerant Molecular Engineering for High‐Rate and Ultra‐Long Cycle Life Zinc Anode
Source: Adv Sci (Weinh). 2025 Jul 2;12(37):e08628. doi: 10.1002/advs.202508628 (PMC12499434; doi:10.1002/advs.202508628)
Supplement: Supplementary file 1 — Supporting Information [file ADVS-12-e08628-s001.docx]

Supporting Information

**Tolerant Molecular Engineering for High-Rate and Ultra-Long Cycle Life Zinc Anode**

*Chenyang Zhao^a^†, Zeping Liu^a^†, Yu Zhang^b^*, Pengyu Wang^a^, Man Qi^b^, Zhikun Guo^a^, Zeen Wu^b^, Xin Zhang^b^, Xingyuan Lu^a^, Jiayin Yuan^b^*, Naiqing Zhang^a^**

*^a^ The State Key Laboratory of Urban-rural Water Resources and Environment, School of Chemistry and Chemical Engineering, Harbin Institute of Technology, Harbin 150001, China*

*E-mail: zhangnq@hit.edu.cn*

*^b^ Department of Chemistry, Stockholm University, Stockholm 10691, Sweden*

*E-mail: yu.zhang@su.se & jiayin.yuan@su.se*

*^†^ These authors contributed equally: Chenyang Zhao, Zeping Liu.*

1. Experimental Section

**1.1 Material synthesis.**

(S)-2-Methyl-1,4,5,6-tetrahydropyrimidine-4-carboxylic acid (PyrB, 99%) was purchased from Guangzhou Shiming Chemical Co. Ltd (Guangzhou, China). Zinc sulfate heptahydrate (ZnSO_4_·7H_2_O), poly(1,1-difluoroethylene) (PVDF, 99%), N-methyl-2-pyrrolidone (NMP, 99%), manganese sulfate (MnSO_4_·H_2_O, 99%), ammonium peroxydisulfate ((NH_4_)_2_S_2_O_8_, 98%) and ammonium sulfate ((NH_4_)_2_SO_4_, 99%) were purchased from Aladdin Bio-chem technology Co. Ltd (Shanghai, China). All the materials were used as received without further purification.

**1.2 Preparation of electrolytes with different PyrB concentrations**

A certain mass of PyrB powder was individually dissolved in 3M ZnSO_4_ electrolyte to obtain a series of PyrB/ZnSO_4_ electrolytes at specific PyrB concentrations in the range of 0.01 M~0.75 M.

**1.3 Preparation of** **PyrB@Zn for characterizations.**

1000-grit sandpaper was firstly used to polish the surface of the zinc foil and then 10000-grit sandpaper was used to finely polish the zinc foil, efficiently removing the oxide layer and obtaining a smooth zinc foil. Symmetric zinc cells were assembled with PyrB/ZnSO_4_ electrolytes and 3 M ZnSO_4_ as a control, using 12.0 mm Zn foil and Whatman GF/A separator. After assembly, the battery was tested on the battery testing system (NEWARE). The PyrB will be electrostatically adsorbed onto the zinc anode, providing protection. After testing, the battery was disassembled, and the electrodes were rinsed with deionized water to remove residual electrolyte, then dried at room temperature to obtain the PyrB@Zn for characterization.

**1.4 Preparation of cathode.**

α-MnO_2_ was synthesized according to the previous literature (Advanced Functional Materials, 2025, 35(1): 2411582.). 3.0 mmol V_2_O_5_ and 2.0 mmol zinc acetate were dissolved in 70 mL deionized water and stirred for 20 min. Then, 2 mL of 10 *wt*% HNO₃ and 5 mL acetone were added with continued stirring. The mixture was transferred to a reactor for hydrothermal treatment at 180 °C for 24 h to finally obtain zinc-intercalated vanadium oxide cathode. The cathode was prepared by mixing 70 wt% of the as-prepared active materials, 20 wt% carbon black, and 10 wt% PVDF, stirred in NMP for 12 hours to form a homogenous slurry. Then, the slurry was coated on stainless steel mesh and dried in a vacuum at 80 ℃ for 12 hours to obtain the cathode.

**2. Material Characterizations.**

The morphologies of Zn and PyrB@Zn were investigated by a Hitachi SU8010 scanning electron microscope (SEM) at 15 kV. A PAN analytical X'Pert PRO X-ray diffraction (XRD) instrument was devoted to characterizing the crystal structures of all samples with a Cu Ka radiation operating at 40 mA and 40 kV. The elemental composition and content were analyzed by X-ray Photoelectron Spectroscopy (XPS) through ThermoFisher ESCALAB 250Xi. The NMR spectra were obtained by AS 400. The principle of fluorescent labeling is based on the characteristics of FITC molecules and their interaction with the target substances. FITC, as a green fluorescent dye, has good fluorescence properties and reactivity. Its excitation wavelength is usually in the range of 490-495 nm, and its emission wavelength is in the range of 515-520 nm in the form of green fluorescence.

**3. Electrochemical Measurements**

All charge and discharge tests were conducted on the NEWARE BTS-610 (Neware Co. Ltd, China) battery tester. Electrochemical impedance spectroscopy (EIS, 10^-2^-10^5^ Hz), cyclic voltammetry (CV) data, linear sweep voltammetry (LSV), Tafel plot and chronoamperometry (CA) curves were collected on a CHI660E electrochemical workstation. The thickness of the zinc foil is 100 μm, and the amount of electrolyte used is 100 μL. For symmetrical battery testing, 1 mAh cm^-2^ and 5 mAh cm^-2^ correspond to zinc utilization rates of 1.7% and 8.5%, respectively. For a full battery, based on actual specific capacity and load, the cycling capacity is approximately 2.28 mAh cm^-2^, corresponding to a zinc utilization rate of 3.9%. The full battery N/P is approximately 25.

**4. Theoretical Calculations.**

The first-principles calculations were performed with the Cambridge Sequential Total Energy Package (CASTEP) based on the density functional theory (DFT). The general gradient approximation (GGA) with the Perdew-Burke-Ernzerhof (PBE) formulation for exchange-correlation functions were utilized in the present calculations. The convergence tolerance for geometry optimization was set as 1.0 × 10^−5^ V for energy, and all the forces on each atom were smaller than 0.02 eV Å^−1^. The cutoff energy was set 450 eV for the plane-wave basis set. The k-points for the Brillion zone were selected by Monkhorts–Pack method and set to 5 × 5 × 1 for Zn (001). The binding energy (E_a_) for each model interacting with a Zn ion was defined as

E_a_= E_total_− E_sub_− E_mol_

where E_total_ is the total energy of the Zn (001)/Zn, Zn (001)/H_2_O, Zn (001)/PyrB-O, Zn (001)/PyrB-N model, E_sub_ is the energy of the Zn (001) model, and E_mol_ is the energy of adsorbed small molecules, respectively.

Molecular dynamics (MD) simulations: The geometric optimization of PyrB, H_2_O and Zn^2+^, SO_4_^2-^ were carried out in the DMol3 module using the local density approximation and GGA with PBE formulation for the reagent molecular optimization, where the SCF tolerance was 10^-5^ eV. Interaction models were established with the Amorphous Cell module. The COMPASS II force field parameters were used to describe the interactions between Zn^2+^, PyrB and H_2_O. A reference modeling system consisting of 30 ZnSO_4_ ion pairs and 660 water molecules was constructed, and a considerable amount of PyrB was introduced corresponding to its concentration of 0.1M in real experiments. Simulation of dynamic properties was conducted in the NVT ensemble, utilizing the Nose thermostat at a temperature of 298 K. The calculation period spanned a total of 1000 ps. The cutoff radius and the electrostatic interactions were both set to 12.5 Å. The Ewald summation method was used for the computation of electrostatic interactions, and van der Waals interactions were computed using the atom-based method. Atomistic simulations of this modeling system were performed using Forcite package with cubic periodic boundary conditions.


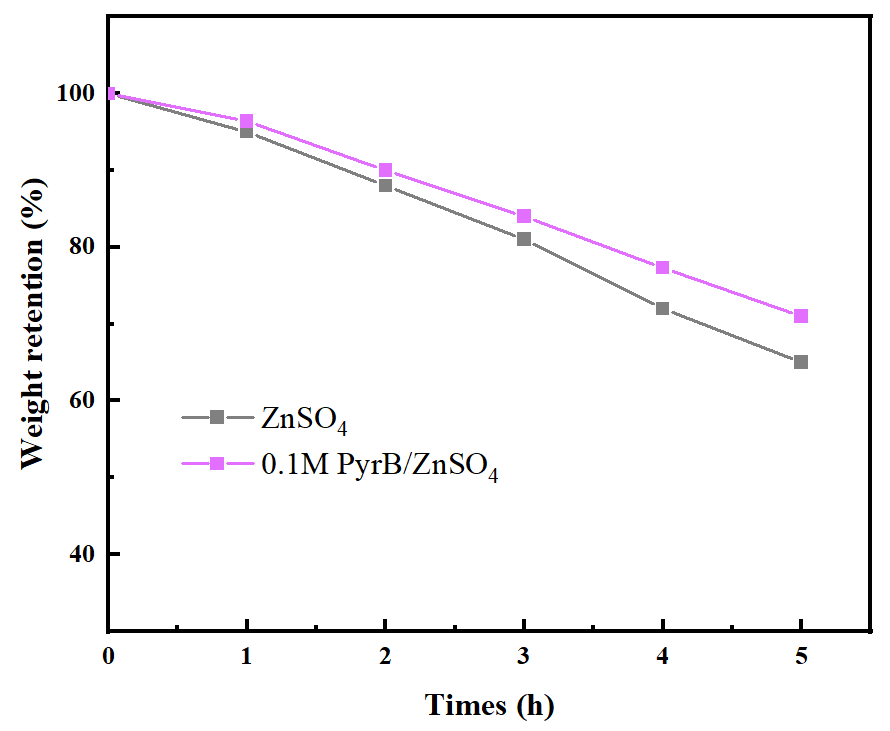


**Figure S1.** The plot of the quality retention rate of two solutions (without and with PyrB) at 60 ℃.


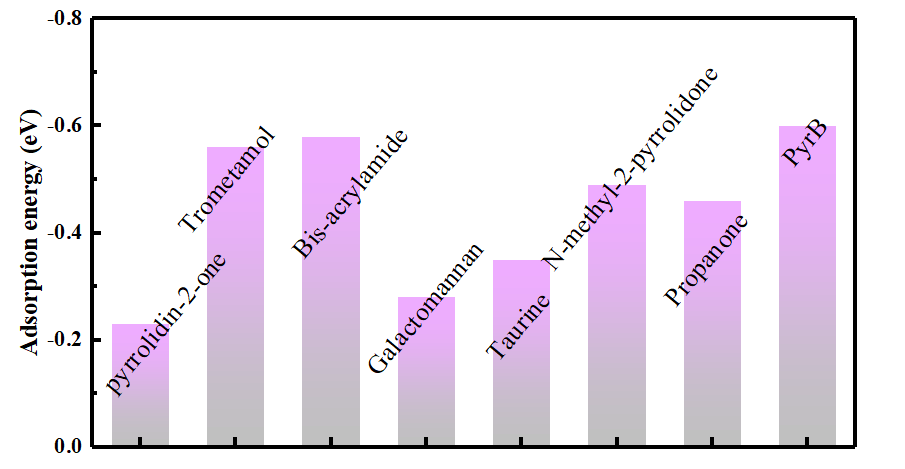


**Figure S2.** Comparison of the adsorption energy of different organic molecules on zinc anode.


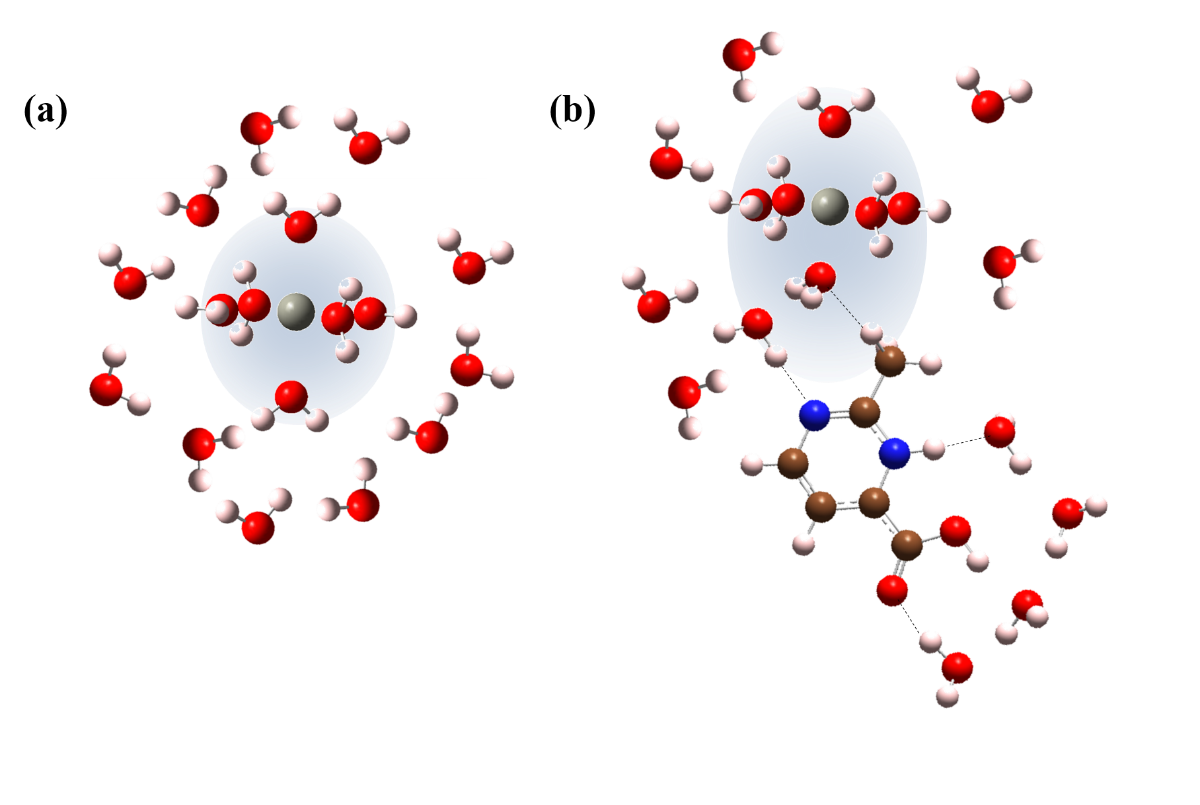


**Figure S3.** Structure diagram of Zn^2+^ solvation (a) in ZnSO_4_ electrolyte; (b) in ZnSO_4_ electrolyte with PyrB added.





**Figure S4.** Bar charts of ion conductivity at different PyrB concentrations.


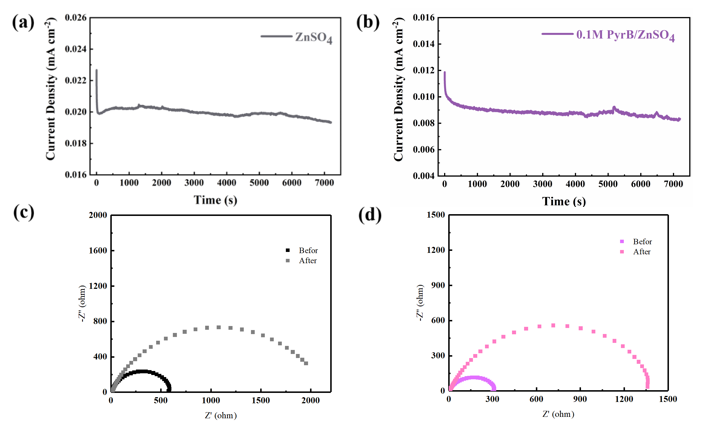


**Figure S5.** The chronoamperometry curves and EIS plot of various zinc anodes (a,c) bare zinc anode; (b,d) PyrB modified Zn anode.





**Figure S6.** Chronoamperometry curves of Zn nucleation on Zn anodes with or without PyrB layer under -150 mV within 100 s.


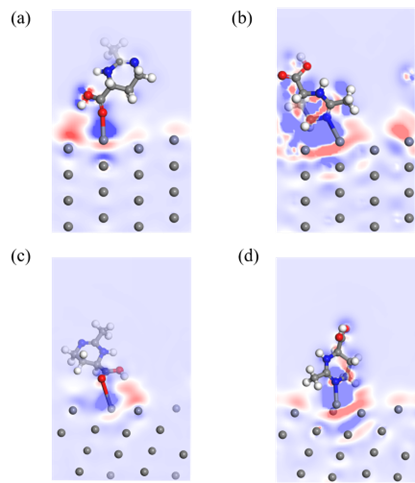


**Figure S7.** The sliced 2D contour map of different charge densities of PyrB molecules at varied sites on different crystal planes of zinc. (a) O site on Zn(100); (b) N site on Zn(100); (c) O site on Zn(101); (d) N site on Zn(101).


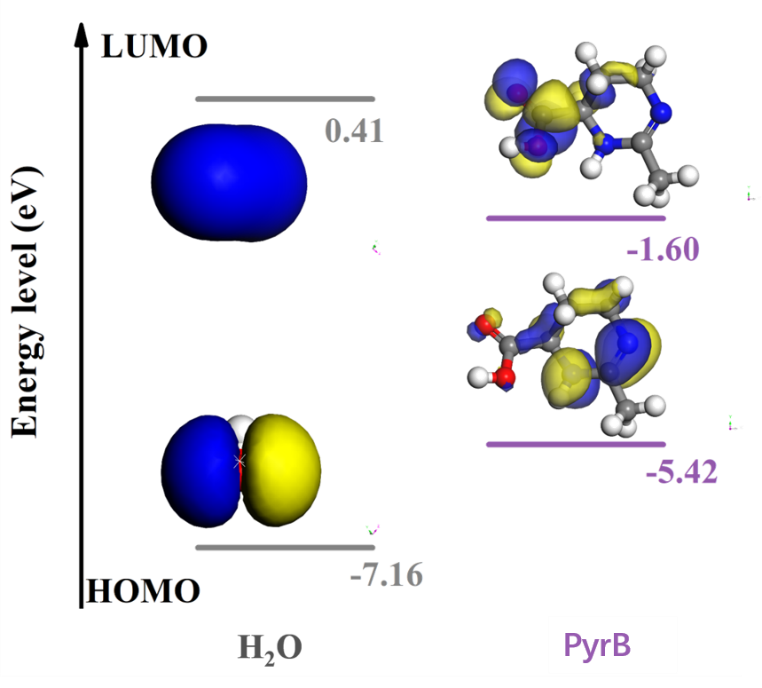


**Figure S8.** Calculated HOMO and LUMO energy levels of H_2_O and PyrB.


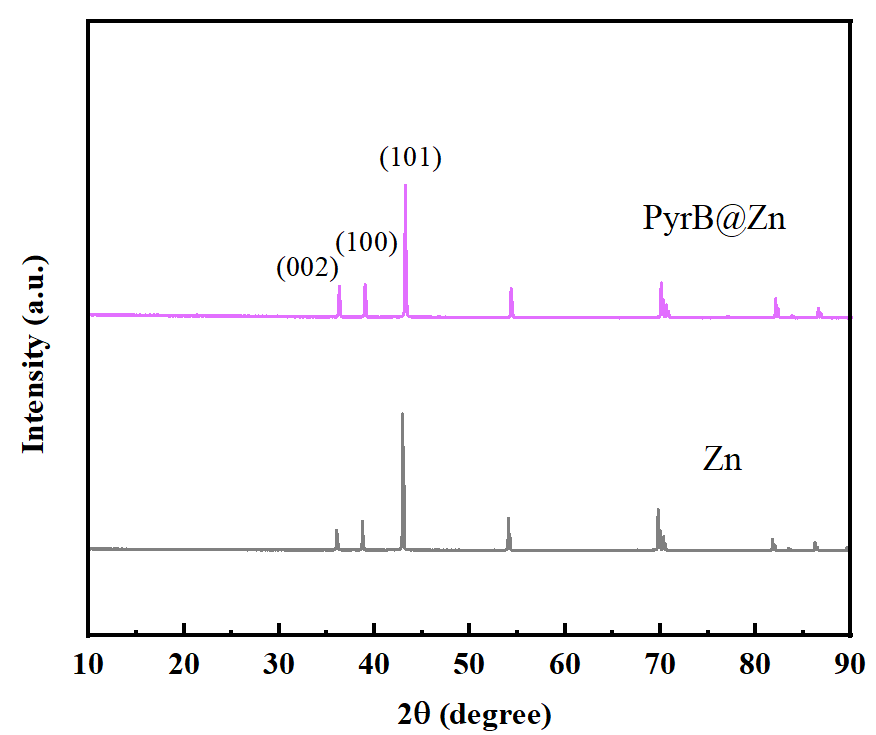


**Figure S9.** XRD spectra of the zinc foil and the PyrB@Zn electrode after zinc deposition at 1.0 mA cm^-2^ and 5.0 mAh cm^-2^.


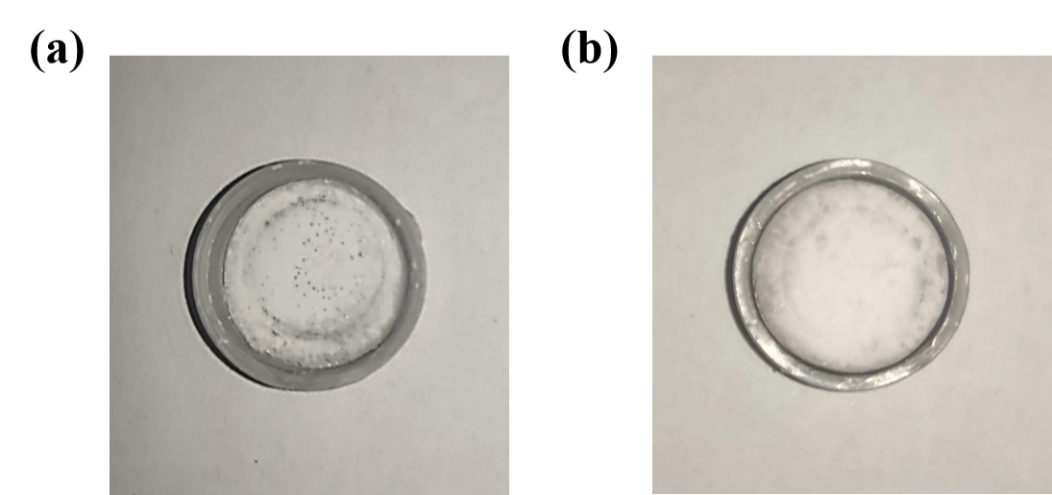


**Figure S10.** Optical images of the separator after cycles of different batteries. (a) bare Zn cell; (b) similar cell with added PyrB.


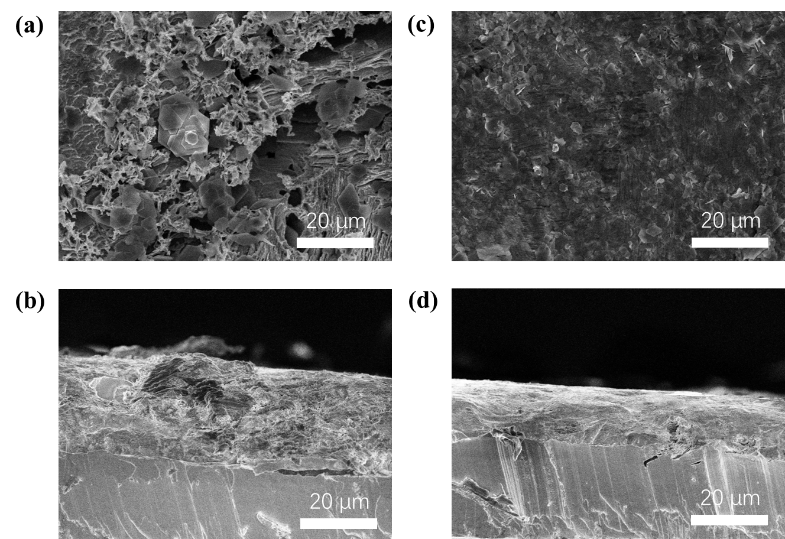


**Figure S11.** (a) SEM plan view and (b)cross-section of the Zn electrode after 50 cycles. (c) SEM plan view and (d)cross-section of the PyrB@Zn electrode after 50 cycles.


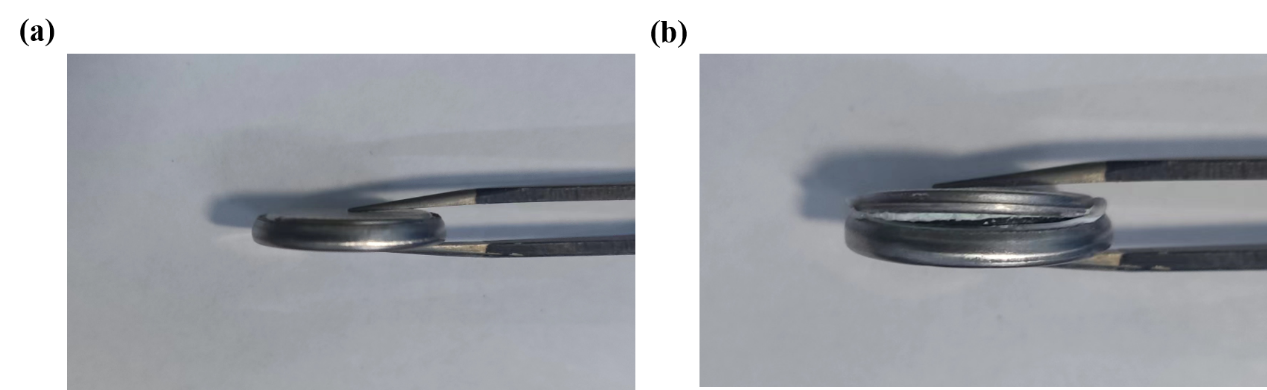


**Figure S12.** Optical images of the cells after cycling. (a) PyrB@Zn cell; (b) bare Zn cell.


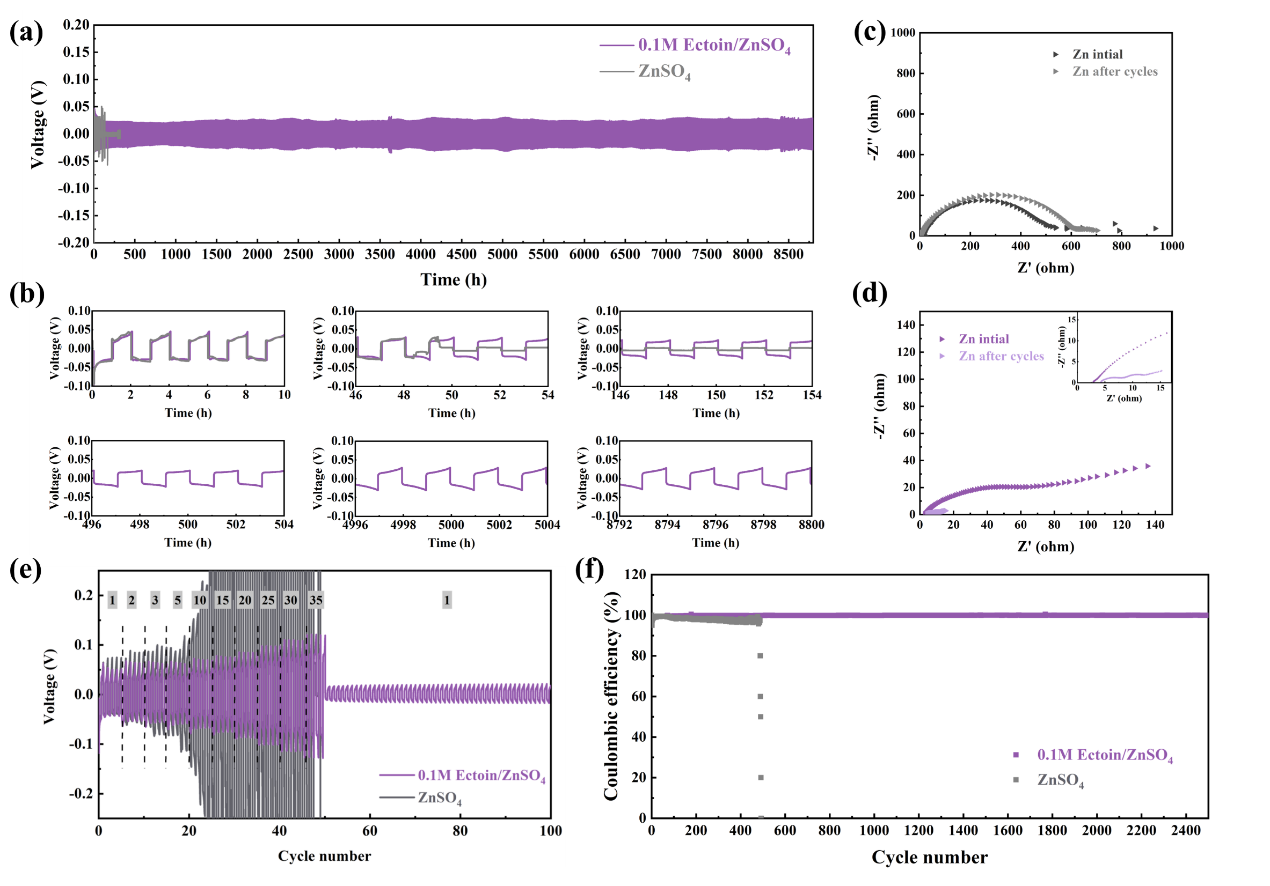


**Figure S13.** Time voltage curves for different times.





**Figure S14.** Cycling stability of zinc symmetric cells at the current density of 10.0 mA cm^-2^ with an areal capacity of 1.0 mAh cm^-2^.





**Figure S15.** Cycling stability of zinc symmetric cells at 20.0 mA cm^-2^ and 5.0 mAh cm^-2^.


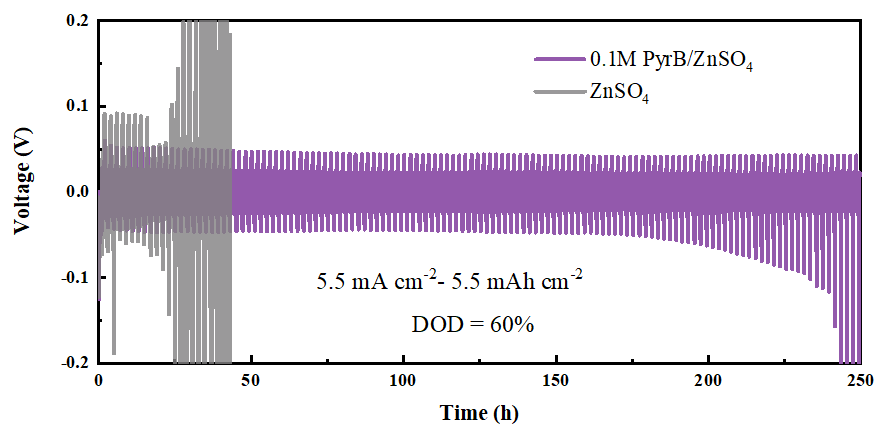


**Figure S16.** Battery cycling performance under high zinc utilization conditions.


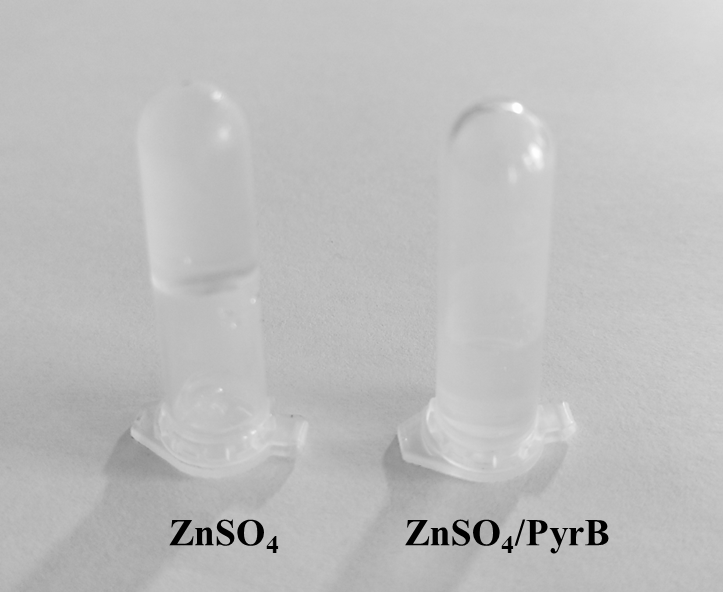


**Figure S17.** Optical photographs of two electrolytes at -10℃.


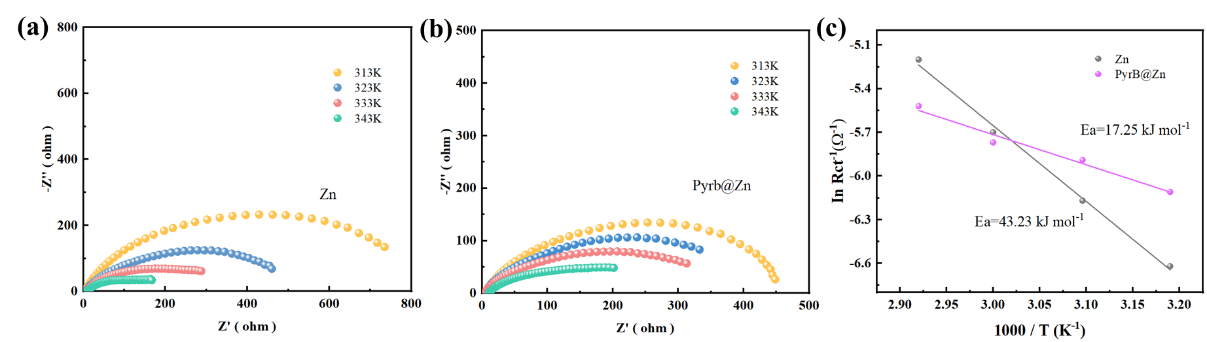


**Figure S18.** (a) EIS spectra of Zn symmetric cells under different temperatures. (b) EIS spectra of PyrB@Zn symmetric cells under different temperatures. (c) The fitted activation energy curve.





**Figure S19.** CE performance of Zn||Cu cells at current density of 5.0 mA cm^-2^ and areal capacity of 1.0 mAh cm^-2^.


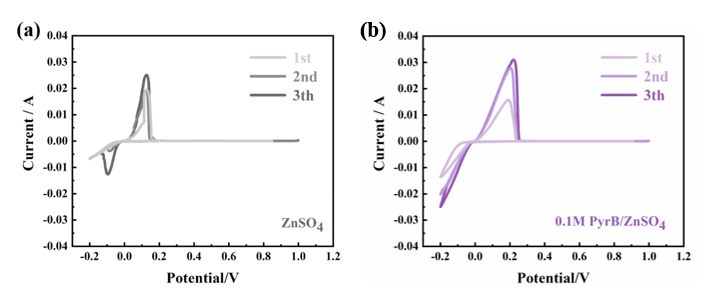


**Figure S20.** Cyclic voltammetry (CV) curves of Zn plating/stripping in PyrB and Zn at a scan rate of 0.5 mV s^-1^ with a potential range of -0.2 to 1.0 V.


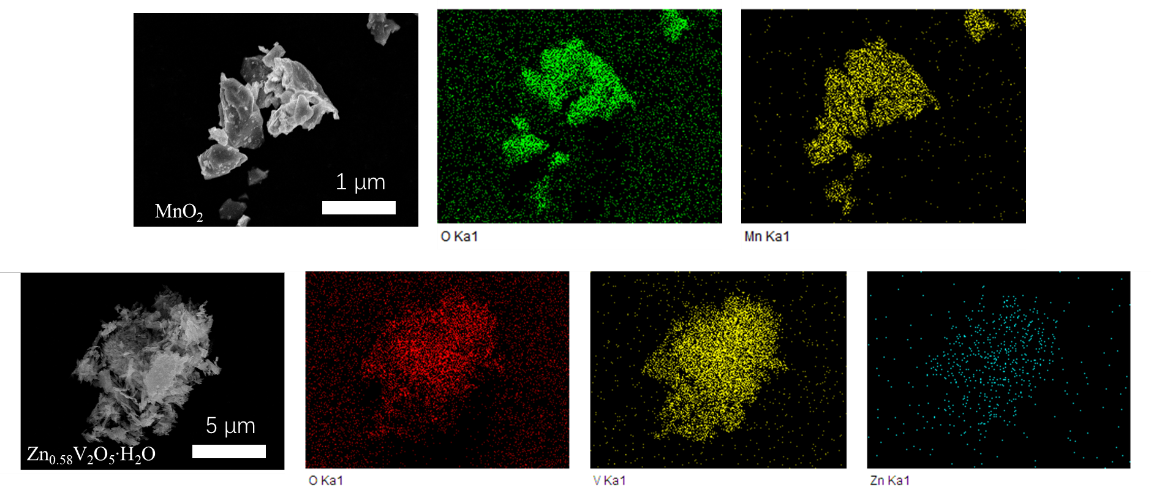


**Figure S21.** Regarding the morphology and corresponding mapping of manganese oxide and vanadium oxide cathode materials.


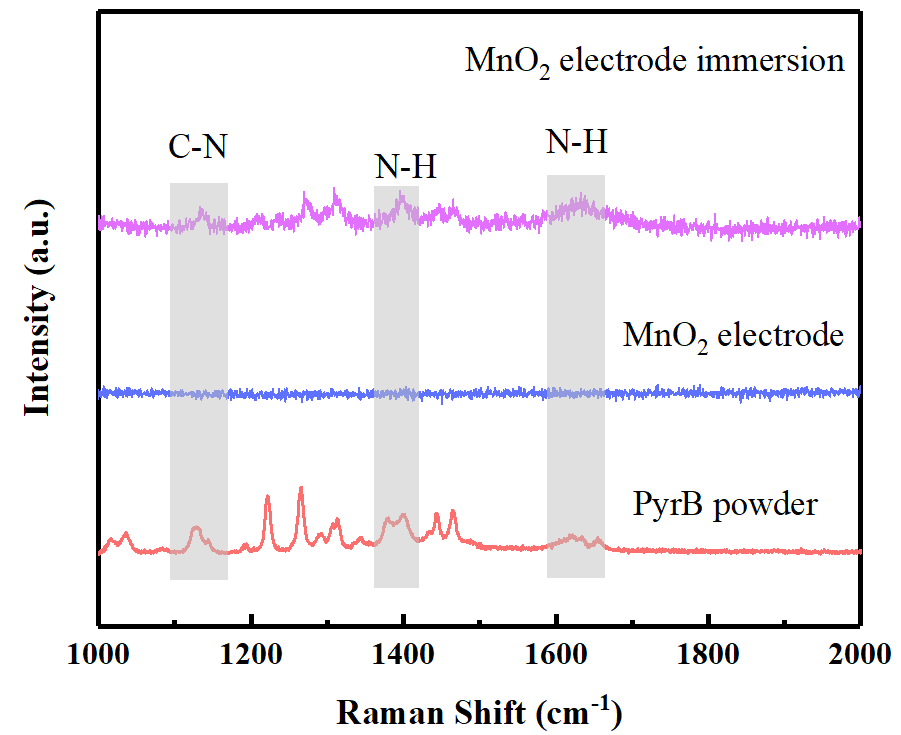


**Figure S22.** Raman spectra after electrode immersion.


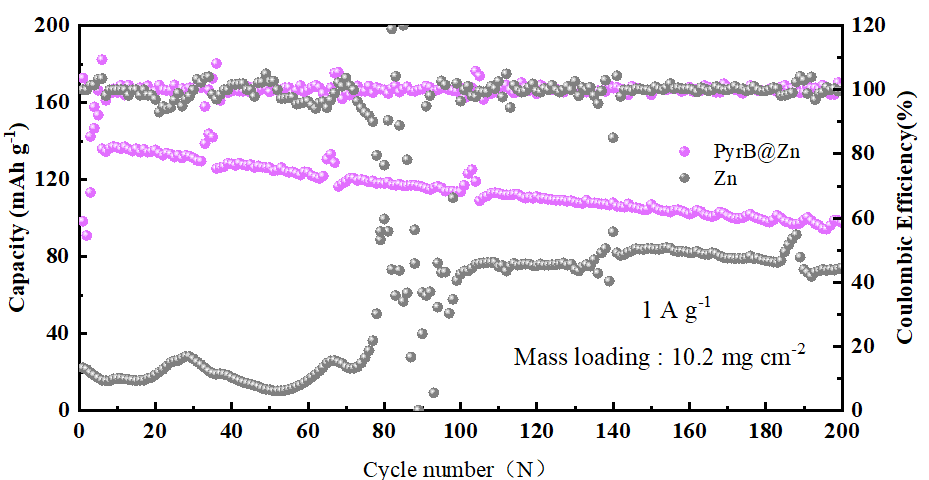


**Figure S23.** The cycling performance of the full cell at -10 ℃.

**Table S1.** A comparison between our work and the reported molecules in zinc-ion batteries

| additives | Current density (mA cm^-2^) | Areal capacity (mAh cm^-2^) | Time (h) | Cumulative plating capacity (Ah) | Reference |
| --- | --- | --- | --- | --- | --- |
| PyrB | **1** | **1** | **8800** | **4.4** | **This work** |
|  | **10** | **5** | **1100** | **5.5** |  |
| Vitamin B_6_ | 1 | 1 | 3200 | 1.6 | *Angew. Chem. Int. Ed.* 2025, 137, e202424255. |
| Ethyl methyl carbonate +methyl acetate | 1 | 1 | 3140 | 1.57 | *J. Am. Chem. Soc.* 2024, 146, 30998-31011. |
| Pyridine nitrogen oxide | 1 | 1 | 2300 | 1.15 | *Adv. Mater.* 2025: 2420489. |
| 3-(hydroxy(phenyl)phosphoryl)propanoic acid | 0.5 | 0.5 | 3000 | 0.75 | *Energy Environ. Sci.* 2024,17, 3443-3453 |
| D-mannose+ amphipathic sodium lignosulfonate | 1 | 0.5 | 6400 | 3.2 | *Angew. Chem. Int. Ed.* 2024, 63, e202402327. |
| 1-butyl-3-methylimidazolium methanesulfonate | 1 | 1 | 2700 | 1.35 | *J. Am. Chem. Soc.* 2025, 147, 8523–8533 |
| L-carnitine | 1 | 1 | 6000 | 3 | *Energy Environ. Sci.*  2023, 16, 2684 |
| γ-butyrolactone | 2 | 1 | 5000 | 2.5 | *ACS Energy Lett.* 2023, 8, 1, 31–39 |
| Polymer additive (PMCNA) | 1 | 1 | 4500 | 2.25 | *Angew. Chem. Int. Ed.* 2023, e202314456 |
| Tween-20 | 1 | 1 | 2500 | 1.25 | *Adv. Mater.* 2024, 36, 2312924. |
| Citric acid+ aspartame | 1 | 1 | 4500 | 2.25 | *Adv. Energy Mater.* 2025 2500674. |
